# A core framework of “mountain management–water management–moist city” for sponge city special planning: The case of northwestern China

**DOI:** 10.3389/fpubh.2022.994137

**Published:** 2022-08-23

**Authors:** Ran Wu, Ye Yang, Junchao He, Jiang Zhu

**Affiliations:** ^1^Department of Landscape Architecture, Southwest Jiaotong University, Chengdu, China; ^2^China Urban Construction Design & Research Institute Co., Ltd., Beijing, China

**Keywords:** sponge city, special planning, northwestern China, semi-arid valley city, planning method

## Abstract

Sponge city special planning represents a basis for sponge city construction in China. It has a leading role in problem-oriented, effect-centered comprehensive, and coordinated construction. In accordance with the *Interim Provisions on the Formulation of Sponge City Special Planning*, China has gradually established a standardized method for formulating sponge city special planning based on the “four-water” framework. While this method emphasizes the normalization of planning contents, it has limitations when dealing with regional characteristics. The present paper clarifies the purposes and demands of formulating sponge city special planning for semi-arid valley cities in northwestern China. It furthermore highlights the limitations of the standard method and suggests a three-level optimization, i.e., problem identification, target indicators, and system construction. Based on the framework of “mountain management–water management–moist city,” this paper proposes a set of ideas for formulating sponge city special planning. In this regard, the discussions are based on the formulation practice of Xining.

## Introduction

Cities are facing numerous issues, such as traffic congestion, air pollution, water pollution, and urban storm-water disasters (1–5). In the past 20 years, many countries in the world have developed similar management systems and construction ideas (6), such as Water Sensitive Cities in Australia and Low Impact Design in the United States. To reduce the drainage burden of cities and restore the natural infiltration capacity of urban surfaces, China has advocated for the construction of a “Sponge City” (7, 8). A “Sponge City” refers to a city that is “elastic” in adapting to environmental changes and coping with natural disasters. In other words, on rainy days, it would absorb, store, retain, and purify water. When necessary, it would release the water (9). As an important basis for sponge city construction in China, sponge city special planning has a crucial role in problem-oriented, effect-centered comprehensive, and coordinated construction (10).

At present, over 100 prefecture-level cities have formulated the sponge city special planning. This includes the first and second batch of China's national pilot sponge cities. Moreover, after long-term practice, China has successfully established a standard method for formulating sponge city special planning based on the “four-water” framework. This method is closely centered on water ecology, water environment, water safety, and water resources, hereinafter referred to as the “four-water theory.” It encompasses problem analysis, target indicator system construction, and sponge system construction and emphasizes the normalization and comprehensiveness of planning contents. Finally, while this method is applicable to rainy plain cities, such as Chengdu, and Shanghai, Beijing, it has limitations with unique natural conditions in northwestern China.

Even though Northwestern China occupies 1/3 of the country's national territorial area, it accounts only for 5% of its water resources. This water resource shortage is one of the development problems facing cities in northwestern China. Because of this, some of the major issues in this region have become effectively formulating sponge city special planning, guiding the implementation of sponge city construction, and building ecologically safe cities. Thus, based on relevant previous studies, this paper examines Xining (part of China's second batch of national pilot sponge cities as a typical representative of northwestern China) as a case study. In order to provide a replicable experience for other cities, the study optimizes the “four-water theory” and suggests ways of formulating the sponge city special planning based on the core framework of “mountain management–water management–moist city.”

## Overview of research and practice in China

### Overview of research

According to the *Interim Provisions on the Formulation of Sponge City Special Planning*, research on sponge city special planning encompasses three main areas of interest. First, it determines the natural ecological patterns and defines the scope of protection. Secondly, relevant research deals with constructing a system of planning control indicators such as the annual total runoff control rate, water environment quality, urban waterlogging control, and unconventional water resource utilization. Lastly, studies have dealt with coordination with related special planning, proposing source emission reduction measures, drainage pipeline channels, storage facilities, sewage treatment and reuse, green infrastructure, layouts, water ecology scales, water environment, water safety, and water resources problems, and guaranteeing land for facilities (11).

**Figure 1:**
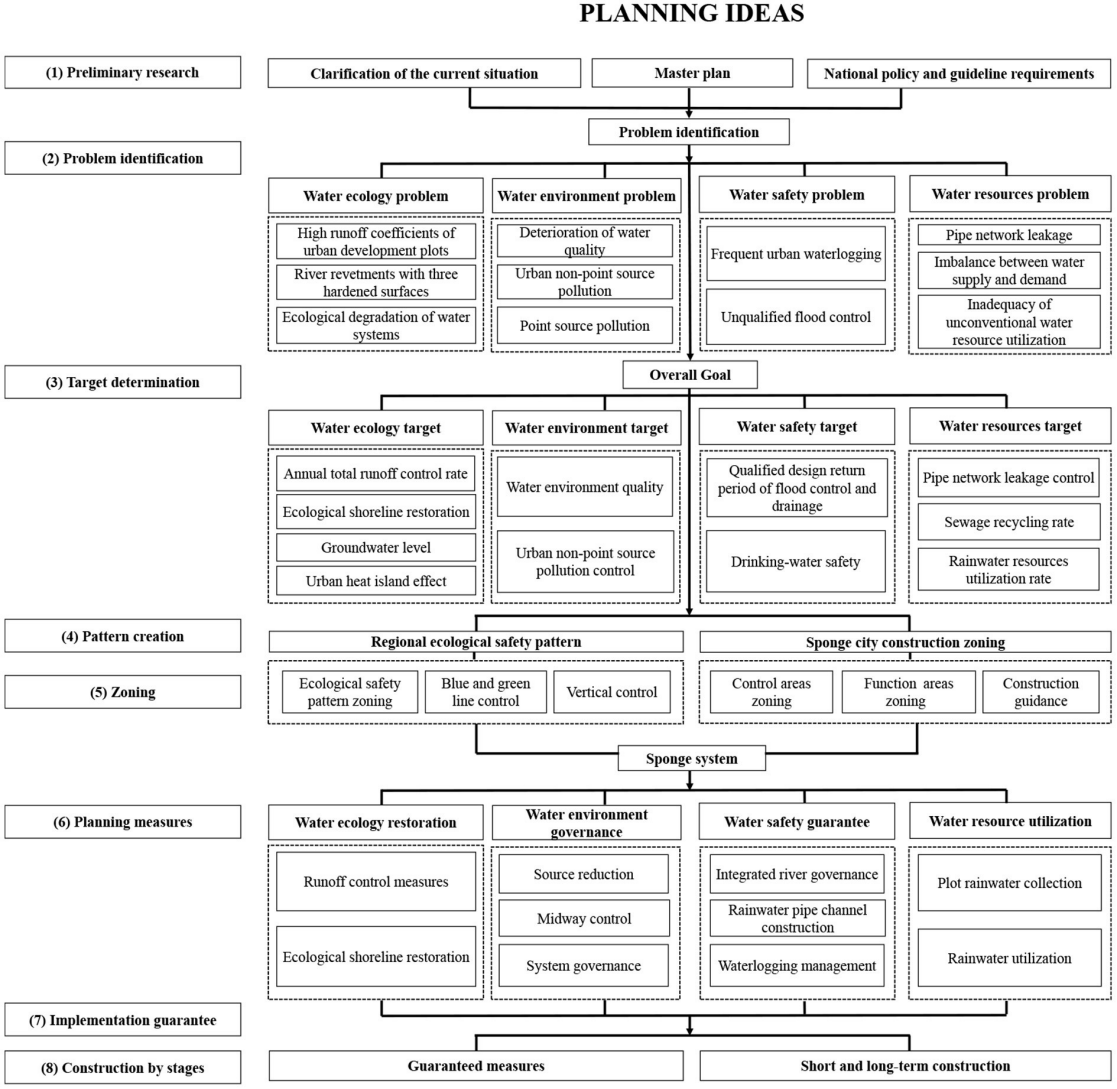
Ideas for formulating the formulation of the sponge city special planning based on the framework of the “four-water theory”.

Research questions regarding sponge city special planning focus on two main aspects. The first aspect involves defining crucial concepts and researching key technologies. This involves explaining major concepts such as sponge city special planning and applying core technologies such as the annual total runoff control rate method. In this regard, Zhang examined the sponge city special planning of Nanning and elaborated on the principles of formulation and the practice of decomposing the target indicator of the annual total runoff control rate (12). The second research aspect relates to sponge city special planning with regard to the “four-water theory.” In this instance, the research object is the planning practice of central urban districts, with the emphasis often placed on how to formulate the sponge city special planning. For example, by examining the practice in Zhuhai, Ma clarified the principles of formulating sponge city special planning and the formulation idea and practice method of the “four-water theory” (10). The “four-water theory” has emerged as the standard method for formulating the sponge city special planning (Figure 1).

With sponge cities being promoted nationwide, improving their planning practicality and feasibility has become a priority in the present stage of sponge city development. Together with examining the characteristics of specific cities, scholars have conducted a series of studies centered on optimizing the “four-water theory” framework. By examining Shanghai Lingang National Pilot Area for Sponge City Construction, Lv et al. (13) proposed that the overall ecological pattern of the city should be protected on the basin scale before implementing the systematic scheme of the “four-water theory”. Furthermore, in view of the special conditions of collapsible loess in Xi'an, Han et al. (14) proposed three sponge layers (large, medium, and small). On the other hand, Yue et al. divided the sponge city special planning types of provincial pilot sponge cities in Sichuan province into three types (water resource utilization type, comprehensive coordination type, and flood disaster management type), and summarized their different planning formulation ideas (15).

### Problems with practice

(1) One-sidedness of contents: As a systematic solution to water problems, sponge cities envelop urban water system planning, water environment improvement planning, flood control and drainage planning, and municipal infrastructure planning. In China, sponge cities are still in the development stage. Some planners lack a systematic understanding of the sponge city special planning and tend to focus on their areas of expertise only. This tendency has given rise to several problems, including one-sided planning contents, inadequate overall planning, and weak practical operability. In practice, planners engaged in municipal water supply and drainage tend to apply existing drainage and flood control planning, focusing only on urban waterlogging risks and ponding points. These planners often leave out systematic issues such as water system ecology, environmental protection, and water recycling and equate the sponge city special planning to drainage and flood control planning, making the systematic role of sponge city special planning in solving related problems impossible.

(2) Homogeneity of achievements: After surveying the formulation of the sponge city special planning in China in recent years, this study observes that the practice tends to adhere to the *Interim Provisions on the Formulation of Sponge City Special Planning* and the “four-water theory” at the levels of problem identification, target determination, and planning measures. There is a lack of concern with transforming theoretical research into practice. Furthermore, regardless of whether the cities face “four-river” problems or if their problems are equally prominent, cities are often treated identically with regard to the construction of the target indicator system and the development of technical measures. As a result, planning formulated in this way fails to match local practical demands and is restricted by its poor operability. For instance, in attempting to solve urban waterlogging problems through rainwater pipe networks, a city in northwestern China imitated the drainage and flood control measures of a southern city. However, as northwestern China has an arid climate and its rainwater facilities have an extremely low utilization rate, these measures result in resource waste. In another case, a rainy southern city set the goal of urban rainwater recycling at 12% (a value that applies only to dry cities facing a prominent contradiction between water supply and demand) in its sponge city special planning. The all-round utilization of rainwater has been stressed by building a lot of new reservoirs and rainwater utilization facilities, causing substantial human and financial losses.

Existing suggestion for formulating the sponge city special planning for semi-arid valley cities in northwestern China is still at the standard method level and is based on the “four-water theory.” In this context, adapting the “four-water theory” to the natural conditions in northwestern China and formulating a sponge city with special planning for this area represents a new research challenge.

## Purposes of formulating the sponge city special planning for semi-arid valley cities in northwestern China

**Figure 2:**
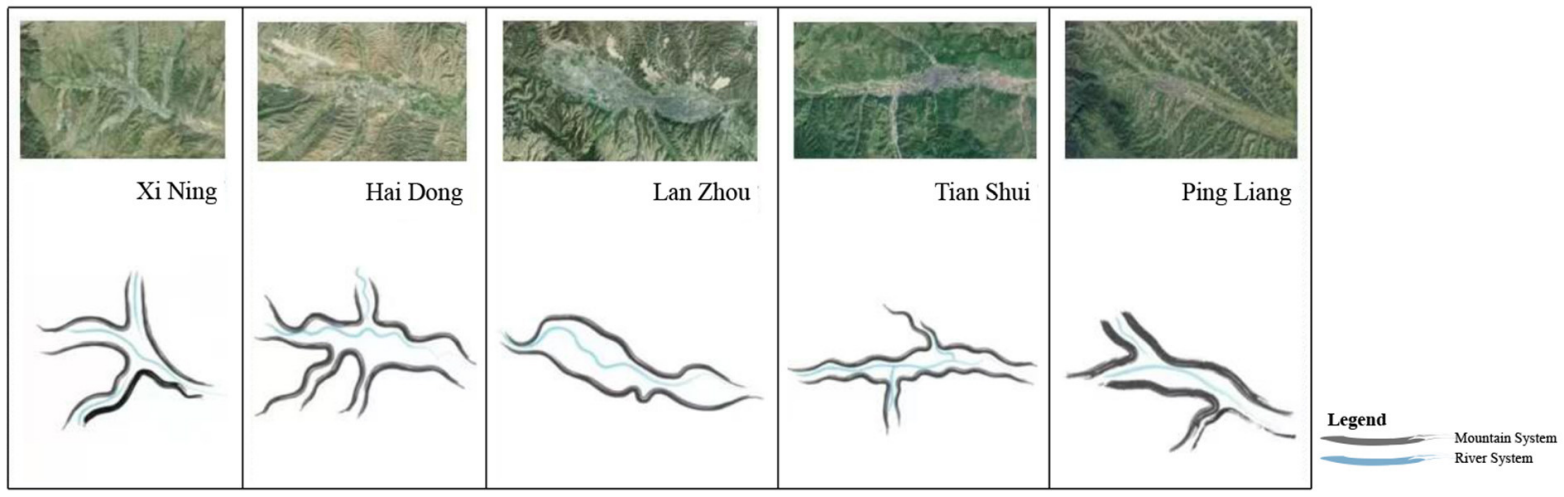
Landscape pattern of semi-arid valley cities in northwestern China.

This paper defines northwestern China's semi-arid valley cities as ones developed in the valleys of the Loess Plateau, which includes southern Shaanxi province, Longnan, Longdong–Ningnan, and Hehuang (Figure 2). As the main force in northwestern China, these cities constitute the development foundation of the region.

Due to the looseness of loess, the urban spaces of these cities rarely extend to the mountains, rather expanding horizontally along the valley terraces. Mountains, water, and city are closely bounded together, presenting the characteristics of “mountains surrounding the city and rivers running through the city.” Typical examples of these cities include Yan'an, Lanzhou, Xining, Haidong, and Tianshui.

The climate of these cities is a semi-arid continental plateau climate, characterized by aridness and a lack of rainfall. The annual average rainfall successively decreases from 500 mm in the east to 300 mm in the west. Furthermore, the annual average evaporation is greater than the annual average rainfall. The per capita water availability of these cities is low, while resources-based and quality-based water shortages coexist (Table 1). Water resources constitute a restricting factor of urban development (16).

**Table 1:** Statistics of rainfall characteristics and per capita water availability in some cities in northwestern China.

**Province**	**City name**	**Rainfall (mm)**	**Evaporation (mm)**	**Per capita water availability Amount**	
				**(m^**3**^)**	**Percentage in national total (%)**
Qinghai	Xining	410	1,212	570	24.78
	Haidong	339	1,644	1,160	50.43
Gansu	Lanzhou	309	1,468	720	31.30
	Tianshui	501	1,277	460	20.00
	Pingliang	481	1,427	683	29.70
	Dingxi	377	1,538	220	9.57
	Qingyang	539	520	350	15.22
Ningxia	Guyuan	426	1,471	372	16.17
Shaanxi	Yan'an	486	950	612	30.22

The regions where these cities are located are heavily affected by soil erosion. The areas of soil erosion are Qinghai, Ningxia, and Gansu and are 46,000 km^2^, 10,000 km^2^, and 134,000 km^2^, respectively. Continuous soil erosion worsens land salinization, resulting in barren land and degraded vegetation. This phenomenon also increases the sediment contents of rivers and lakes, causing river blockage and riverbed elevation. In addition, soil erosion may also easily induce landslides, collapses, debris flows, and other natural disasters, posing constant threats to urban safety.

The seriously polluted and polluted basin area in northwestern China accounts for 40.2% of the total area in this region. The water quality monitoring sections in central urban districts have a compliance rate of about 50%. A total of 14 out of the 28 river sections monitored in Gansu province have reached the standard, accounting for 46.8% of the total monitored area. The main causes of pollution include ever-increasing sewage loads, low urban sewage treatment rates, large industrial wastewater discharge amounts, insufficient eco-environmental water supply, and weak river self-purification capacity.

Based on the above, solving the issues of water shortage, soil erosion, and water environment pollution is urgently needed in sponge city construction for semi-arid valley cities in northwestern China, as these are core issues relevant for formulating sponge city special planning.

## Challenges faced by semi-arid valley cities in northwestern China in formulating the sponge city special planning

After examining the three iterations of revised sponge city special planning by Xining, the study proposes that there are three major challenges in the formulation process:

### Preparing an integrated analysis method for highlighting systematic thinking

Because sponge city special planning resolves cities' water system problems, the systematic analysis method constitutes the basis for scientific planning. The “mountain–water–city” pattern is a typical feature of semi-arid valley cities in northwestern China. The unbalanced spatial pattern caused by the encroachment of high-intensity urban development on water systems and mountains constitutes the root of water problems in such cities. To resolve these issues, the mountain, water, and city need to be treated as a whole; the hydrological process characteristics of the basin should be analyzed; and the urban water problems against the “mountain–water–city” pattern should be examined, while fragmented problem identification needs to be avoided. For instance, analyzing the causes of deteriorating river water quality should include the endogenous pollution of the river itself, the non-point source and point source pollution discharged by the city, and the exogenous pollution discharged into the river by mountain soil erosion. Here, a systematic analysis of the interconnectedness of mountains, water, and the city represents the key to identifying the root of problems.

### How to construct a target-oriented indicator system to adapt to local conditions

Constructing a sound target indicator system with a clear orientation is a prerequisite for promoting sponge city construction. Because of the semi-arid rainless climate and superior vertical drainage conditions in the northwestern region, semi-arid valley cities do not have any prominent water safety problems under a rainfall frequency of ≤ 90% (corresponding to a design rainfall of ≤16.5 mm). In this case, water resource problems are the primary factor restricting urban development. However, the “four-water” problems cannot be lumped under one head. Therefore, the core issue of optimizing the “four-water theory” indicator system, grasp the key points, strengthen the advantages, make up for the shortcomings, and construct a target indicator system satisfying the urgent needs of such cities.

### How to develop systematic measures to avoid the risks of multi-channel construction

**Figure 3:**
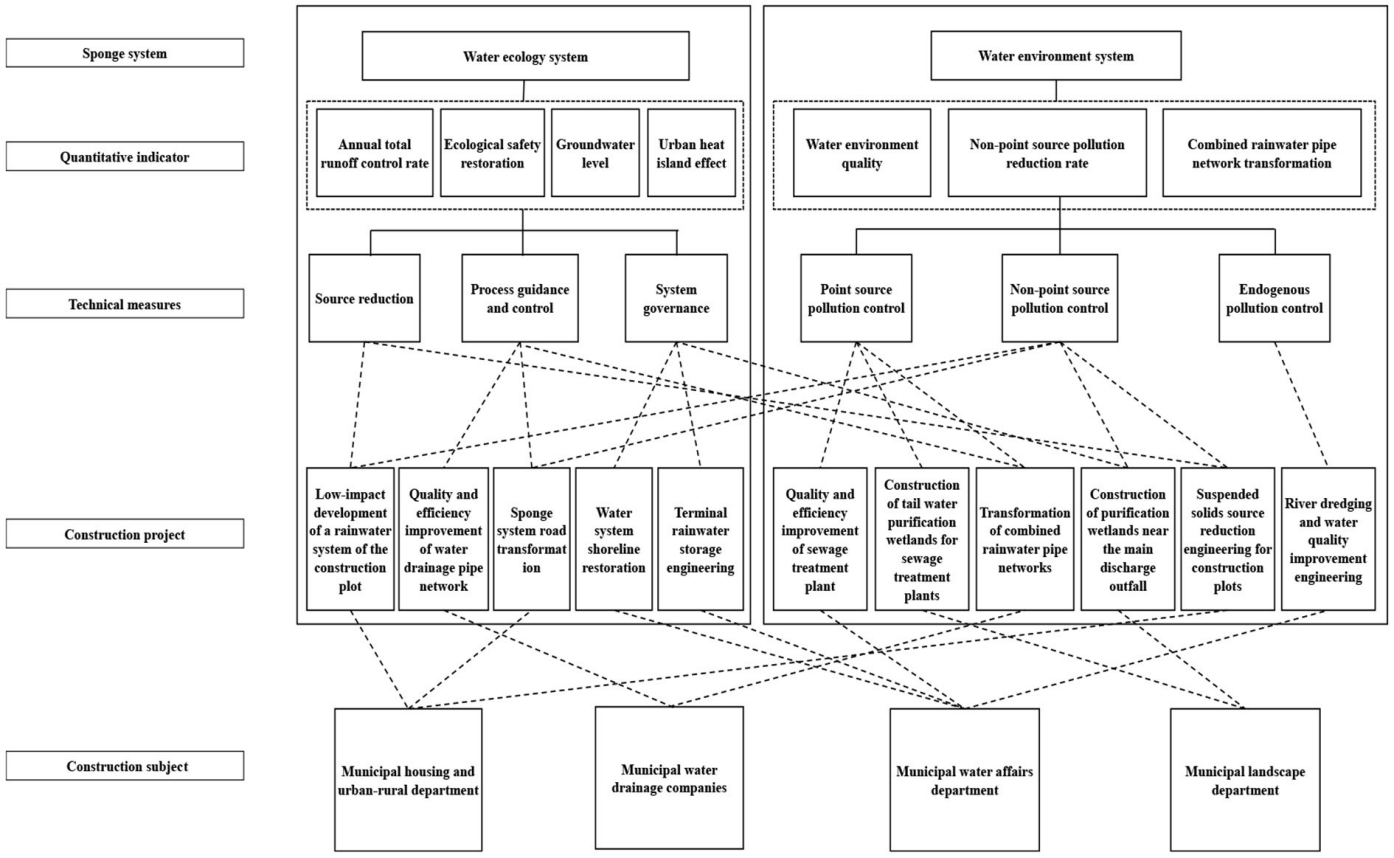
Relationship between “water ecology” and “water environment” systems in the sponge city special planning measures of a city.

The planning measures of the “four-water theory” are closely related. For example, the source low-impact development measures in the “water ecology” system contribute to the non-point source pollution control of the “water environment” system and the development of the “water safety” and “water resources” systems. However, measures are not entirely associated with objectives, projects, and construction subjects, potentially leading to excessive project construction, unclear responsibilities and rights of construction subjects, and poor project management (Figure 3). Because of this, the emphasis of formulating lies in clarifying the “objective–measure–project–construction subject” relationship, developing classified planning measures, and avoiding the risks of multi-channel construction and management. With regard to semi-arid valley cities in northwestern China, it is necessary to examine water ecology, water resources, water environment, and water safety against the “mountain–water–city” pattern. After this, it is crucial to integrate and optimize engineering measures and thus ensure the accessibility of multiple objectives.

## Optimizing the formulation of the sponge city special planning based on the framework of “mountain management–water management–moist city”

**Figure 4:**
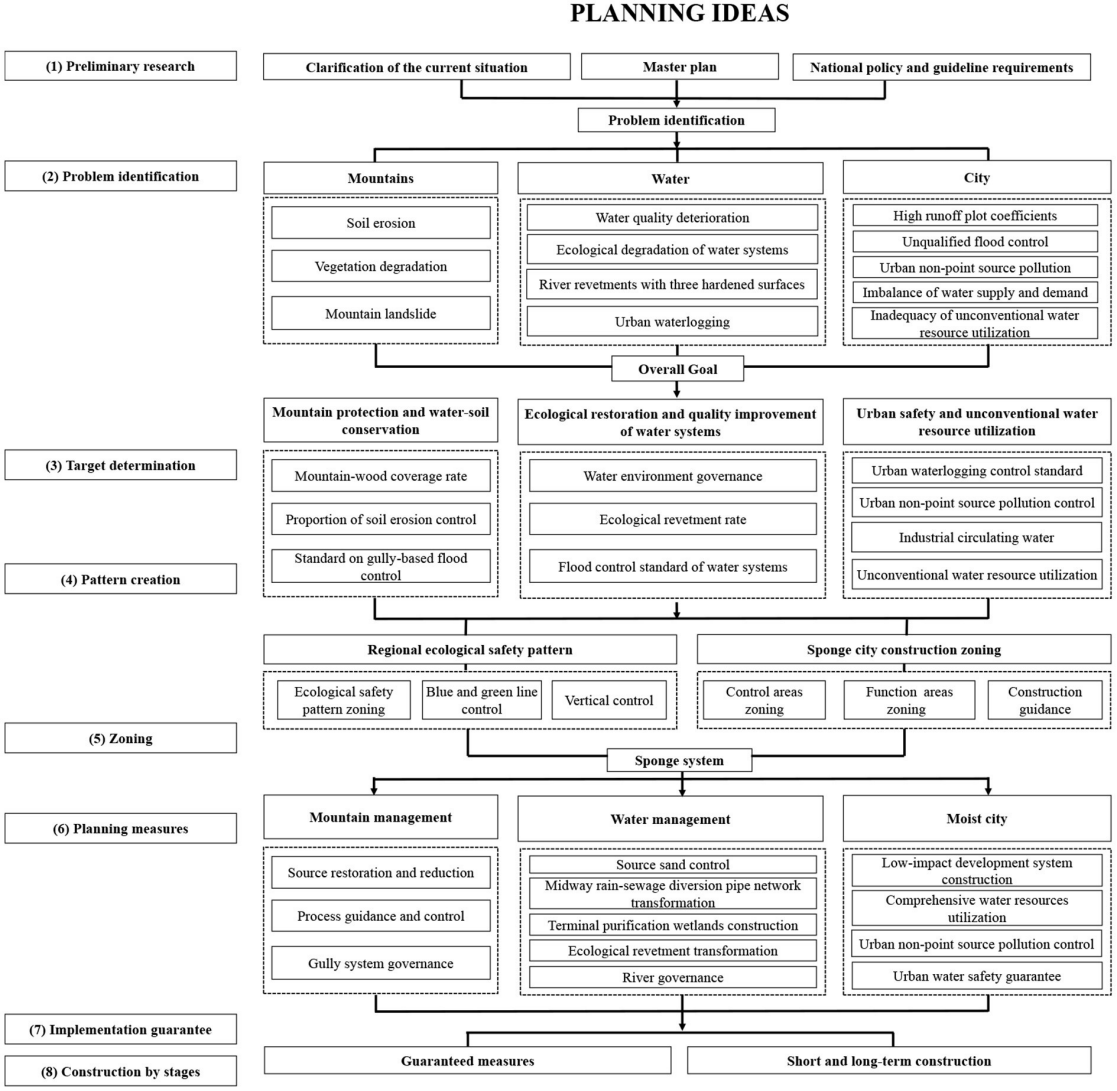
Ideas for formulating the sponge city special planning based on the framework of “mountain management–water management–moist city”.

Based on the “four-water theory” framework, optimization should be provided in terms of problem identification, target determination, and planning measures. This will help to overcome the issues of incomplete coverage or deviation and adapt the “problem–objective–measure” approach to the realities of northwestern semi-arid valley cities (Figure 4).

### Optimization of analysis method

In order to construct a systematic analysis framework of “mountain management–water management–moist city,” this study uses a classification method based on regional characteristics. At the macro level, large spaces such as the mountains outside central urban districts, construction land across urban districts, and the city water systems are systematically analyzed. On the other hand, at the micro level, specific problems of mountains, rivers, and cities are examined, and their causes and inner connections are analyzed.

**Figure 5:**
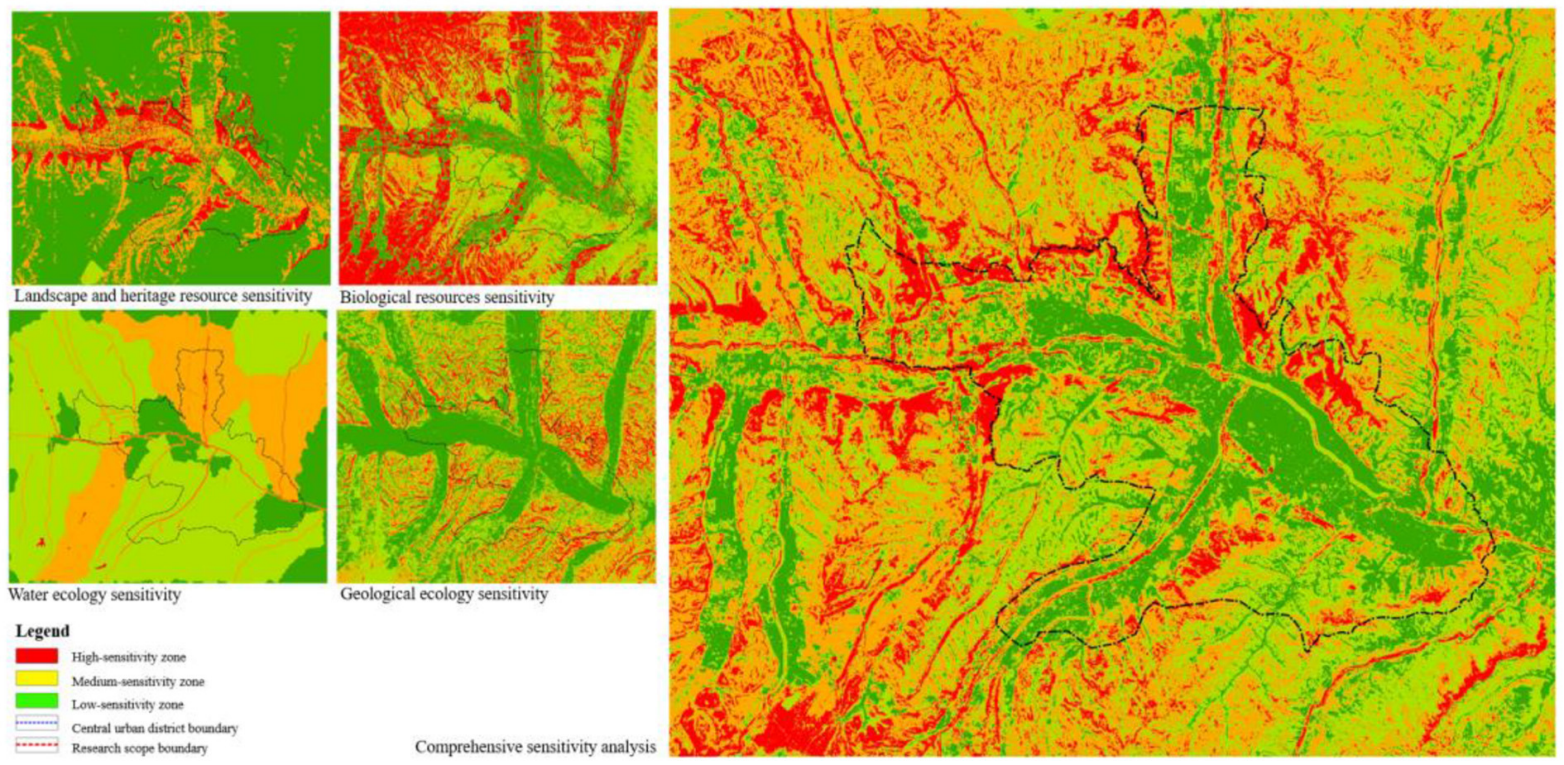
Comprehensive ecological sensitivity analysis of central urban districts in Xining.

**Figure 6:**
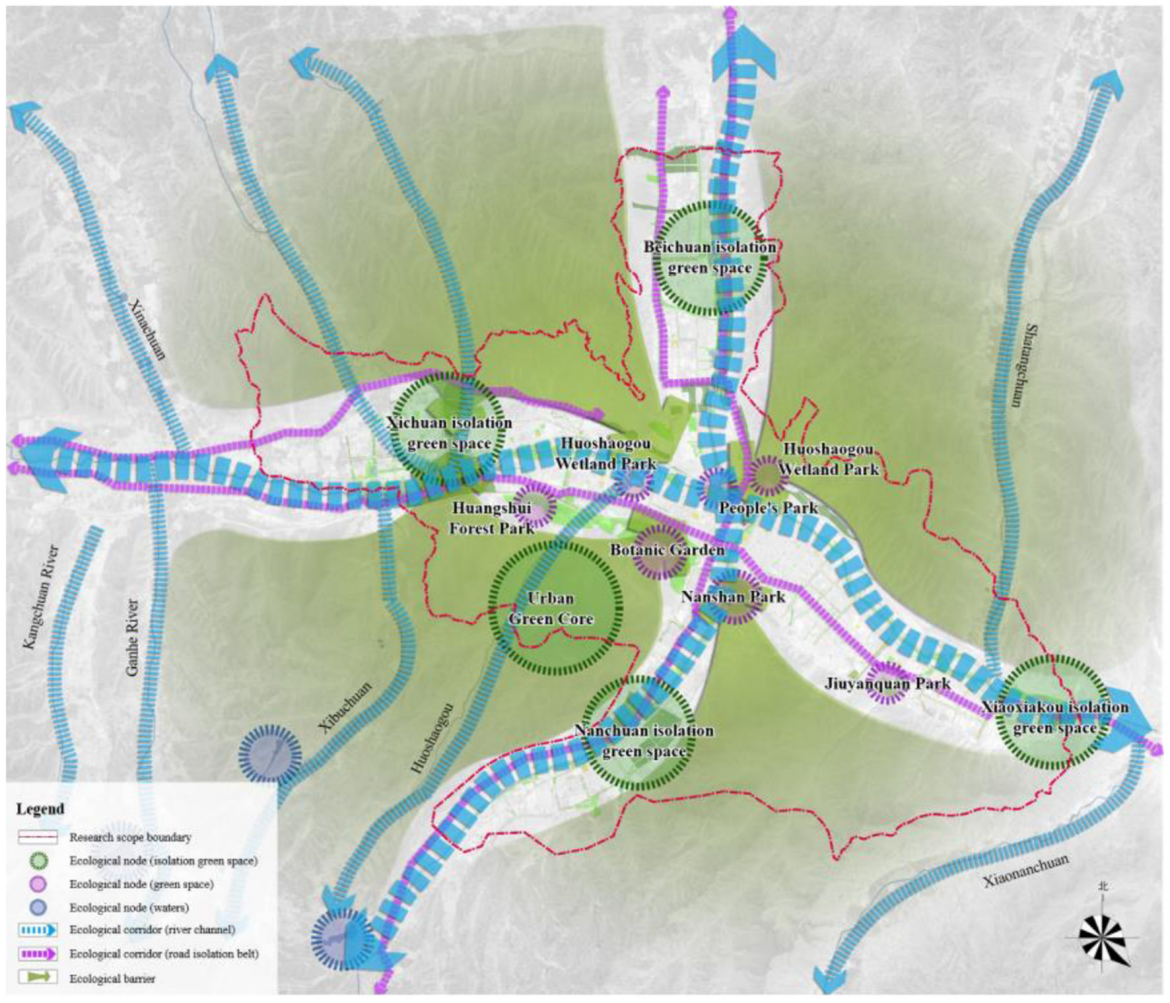
Ecological safety pattern of Xining.

In the case of Xining, the first step is evaluating the suitability of sponge city construction land in central urban districts while also identifying important ecological corridors, large blue and green spaces, and low-lying natural land. After this, an ecological sensitivity analysis should be performed (Figure 5) to build the urban ecological safety pattern (Figure 6) and lay a green foundation for systematic sponge city construction at the regional level. On this basis, the study conducts an analysis centered on mountains, water, and the city.

**Figure 7:**
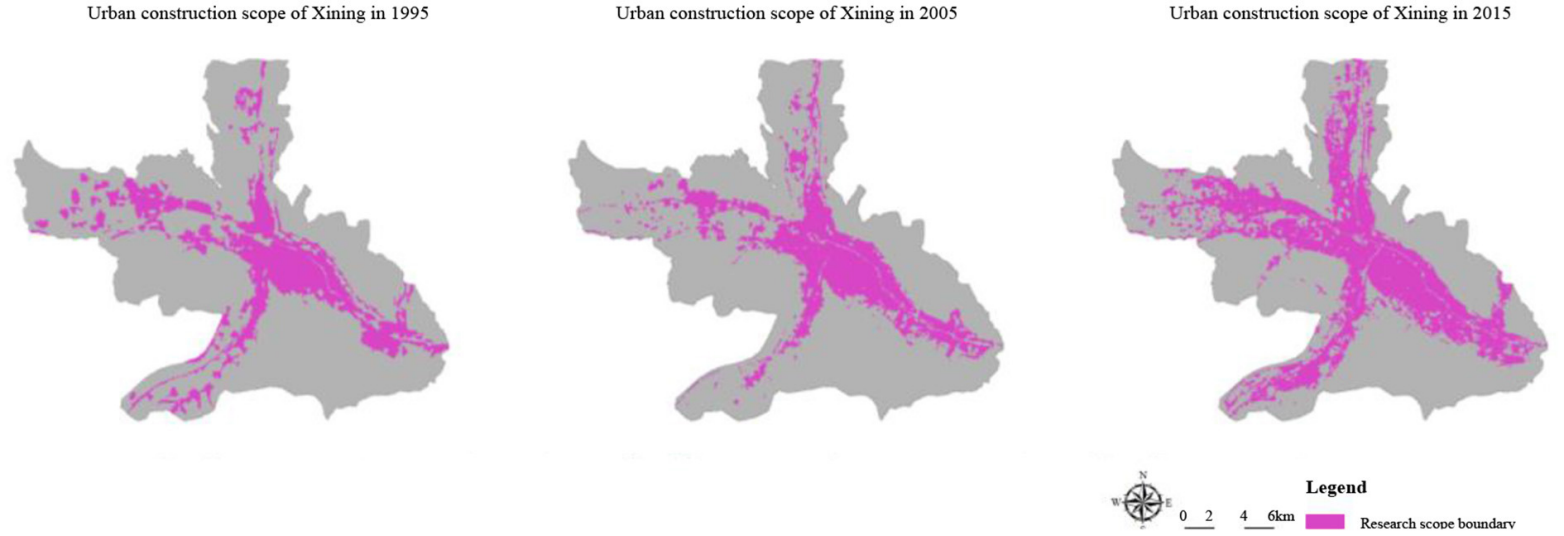
Remote sensing analysis of urban construction land in Xining.

(1) Mountains: safety risks posed by soil erosion and gully erosion. The mountain area amounts to 40% of the central urban districts in Xining. Because of degraded vegetation and sandy soil, Xining is faced with serious soil erosion (mainly surface erosion), only further complicated by gully erosion and gravity erosion, with an erosion modulus of 3,500–5,000 t/(km^2^·a). The area of soil erosion on mountain slopes is about 4.4 km^2^, while that of gully erosion amounts to 70.9 km^2^. Direct threats are posed to urban safety and water system ecology.

(2) City: the shortage of water resources and inadequacy of unconventional water resource utilization. The shortage of water resources in Xining is caused by simultaneous resources and engineering shortages. The average water resources quantity of Xining is 167,000 m^3^/km^2^, making up 2/3 of the national average. The primary problem facing Xining is the disregard for unconventional water resource utilization during a water shortage. On the one hand, from 1995 to 2015, the built-up area of Xining expanded from 68 to 118 km^2^ (Figure 7). Due to high-intensity urban development, the average comprehensive runoff coefficient of central urban districts in Xining rose to 0.51, while the traditional “mode of rapid rainwater drainage” caused resource waste. On the other hand, Xining lacked the facilities necessary to utilize rainwater and reclaimed water, meaning that tap water was still used for greening, irrigation, and road cleaning.

(3) Water: interruption of natural circulation and water pollution above an acceptable level. High-intensity urban development has severed the connection between mountain gullies and river channels and has blocked the natural circulation of water systems. Finally, it has also intensified the risks of gully collapses and debris flows. Hardened river channels isolate the material exchange between soil and water, resulting in the deterioration of river ecology. Revetments with the “three hardened surfaces” (hardened riverbed and hardened banks on both sides) in the central urban districts of Xining have a total length of 55.4% km, accounting for 80% of the total river channel length in the city. According to the water quality of main streams and tributaries in Xining, the mainstream of the Huangshui River is slightly polluted, while its tributaries are seriously polluted. In recent years, the water quality of this river has been maintained at either class V or inferior class V. The major pollutants are NH_4_-N and COD, while the main pollution sources include the non-point source pollution caused by initial rainwater, the exogenous pollution caused by mountain soil erosion, and the point source pollution induced by combined rainwater pipe networks, and the endogenous pollution from internal source pollution stemming from river sludge.

### Optimization of the indicator system

Through a problem-oriented approach centered on urban demands, this paper adds indicators related to mountain protection and restoration based on the “four-water theory” indicator system (Table 2). It further supplements and refines the water resource utilization indicators, weakens the indicators related to urban heat islands and water safety and constructs the “mountain management–water management–moist city” target indicator system (Table 3). In order to guarantee urban safety and river water quality, the proportion of soil erosion control and the standard on gully-based flood control are considered with regard to mountain management.

**Table 2:** Target indicator system based on the “four-water theory” framework.

**Target classification**	**Single indicator**
Water ecology protection	Ecological shoreline proportion of urban inland rivers
	Annual total runoff control rate
	Constant groundwater level
	Urban heat island effect
Water environment governance	Annual SS removal rate
	Transformation rate of combined rainwater pipe networks
	Water quality of river sections in central urban districts
Water safety	Design return period of water drainage system
	Standard on river flood control
	Standard on waterlogging response
Water resource utilization	Rainwater resources utilization rate

**Table 3:** Indicator system for the sponge city construction based on the “mountain management–water management–moist city” framework.

**Target classification**		**Construction indicator**	**Value**
Mountain management	Water conservation	Mountain vegetation coverage rate (added)	≥85%
		Annual total runoff control rate/corresponding design rainfall	98%/27.4 mm
	Soil and water conservation	Proportion of soil erosion control (added)	≥80%
		Standard on gully-based flood control (added)	30 years
Water management	Diversion of clean water into the Huangshui River	Water quality of river sections	Class IV surface water standard or better
		Design return period of river flood control	A-hundred-year return period
	Blue and green intertwining	Ecological shoreline proportion of water systems	≥85%
Moist city	Moist city in mild rain	Annual total runoff control rate/corresponding design rainfall	85%/13.0 mm
		Comprehensive reduction rate of SS	≥50%
	Balance of utilization and drainage	Design return period of rainwater pipe channels	Two-to-five-year return period
		Design return period of waterlogging control	Fifty-year return period
		Proportion of rainwater utilized as a substitute for urban tap water (optimized)	≥2%
		Sewage recycling rate (added)	≥50%

### Optimization of planning measures

Sponge systems classified according to the types of water-related problems are optimized as sponge systems classified according to their regional characteristics. In order to develop technical measures based on co-management, urban sponge systems are constructed from three aspects: mountains, water systems, and cities. Construction projects solve overlapping issues and ensure the “objective–measure–project–construction subject” correspondence.

**Figure 8:**
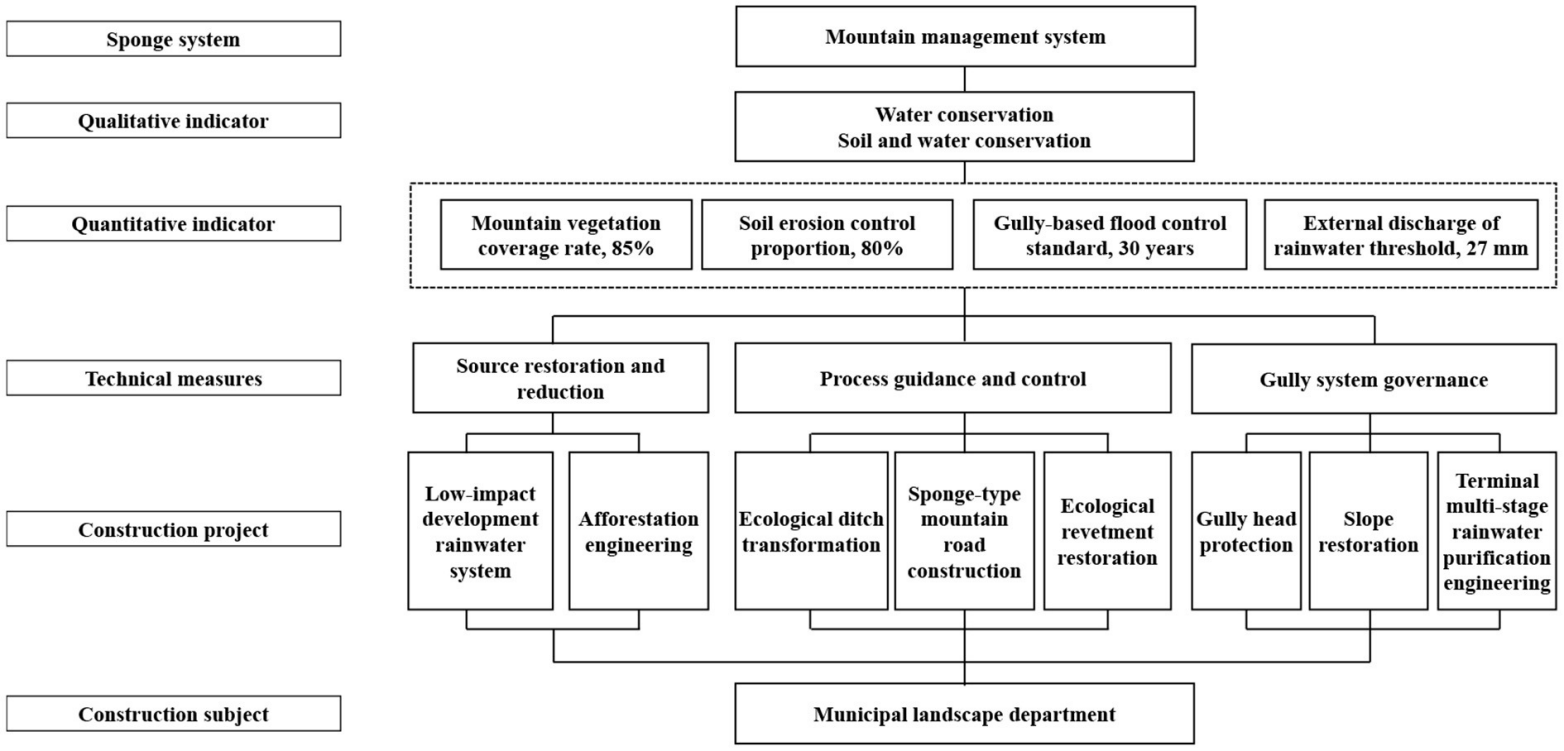
Sponge system for “mountain management” in Xining.

**Figure 9:**
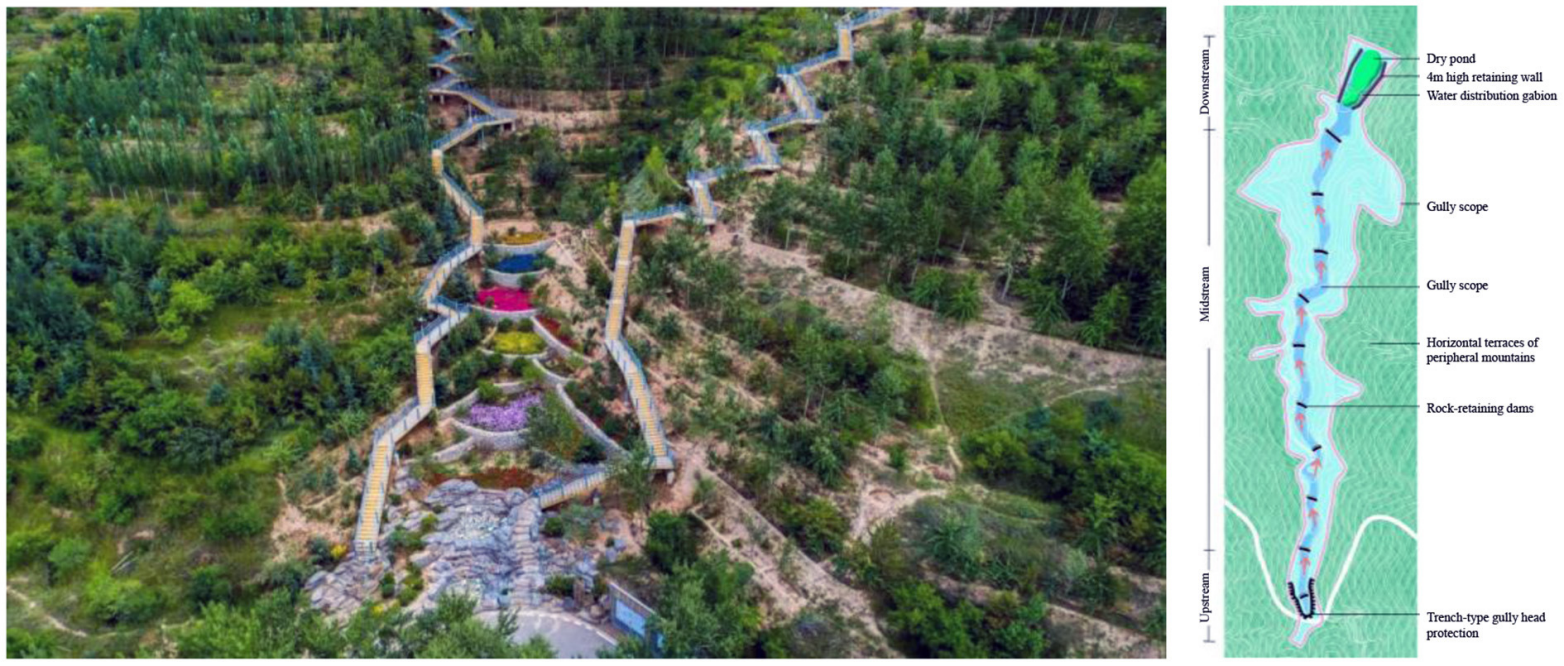
Mountain gully management model and construction effect in Xining.

A “mountain management, water management, and moist city” sponge system was constructed based on the formulation of the sponge city special planning in Xining. The “mountain management” system is centered around the mountains outside central urban districts. It practices the technical measures of source restoration and reduction, process guidance and control, and gully system governance. Finally, this system also establishes green ecological barriers for the city. Forest culture and management and horizontal terrace restoration technologies are employed for the ecological restoration of woodlots, the protection of middle and high-mountain water conservation forests, and the artificial replanting or closed afforestation of sparse natural tree and shrub woods. During these processes, roadside ditches are ecologically transformed, while irrigation water and rainwater runoff are effectively controlled. Small watersheds are taken as units at the system level. At this level of analysis, the double standards on gully-based flood control and geological disaster protection are met through gully head protection, slope restoration, terminal multi-stage rainwater purification, and storage system construction. The construction projects are integrated into eight categories and placed under unified construction and management by the municipal landscape department. Figure 8 shows the sponge system for “mountain management,” while Figure 9 illustrates the construction effect.

**Figure 10:**
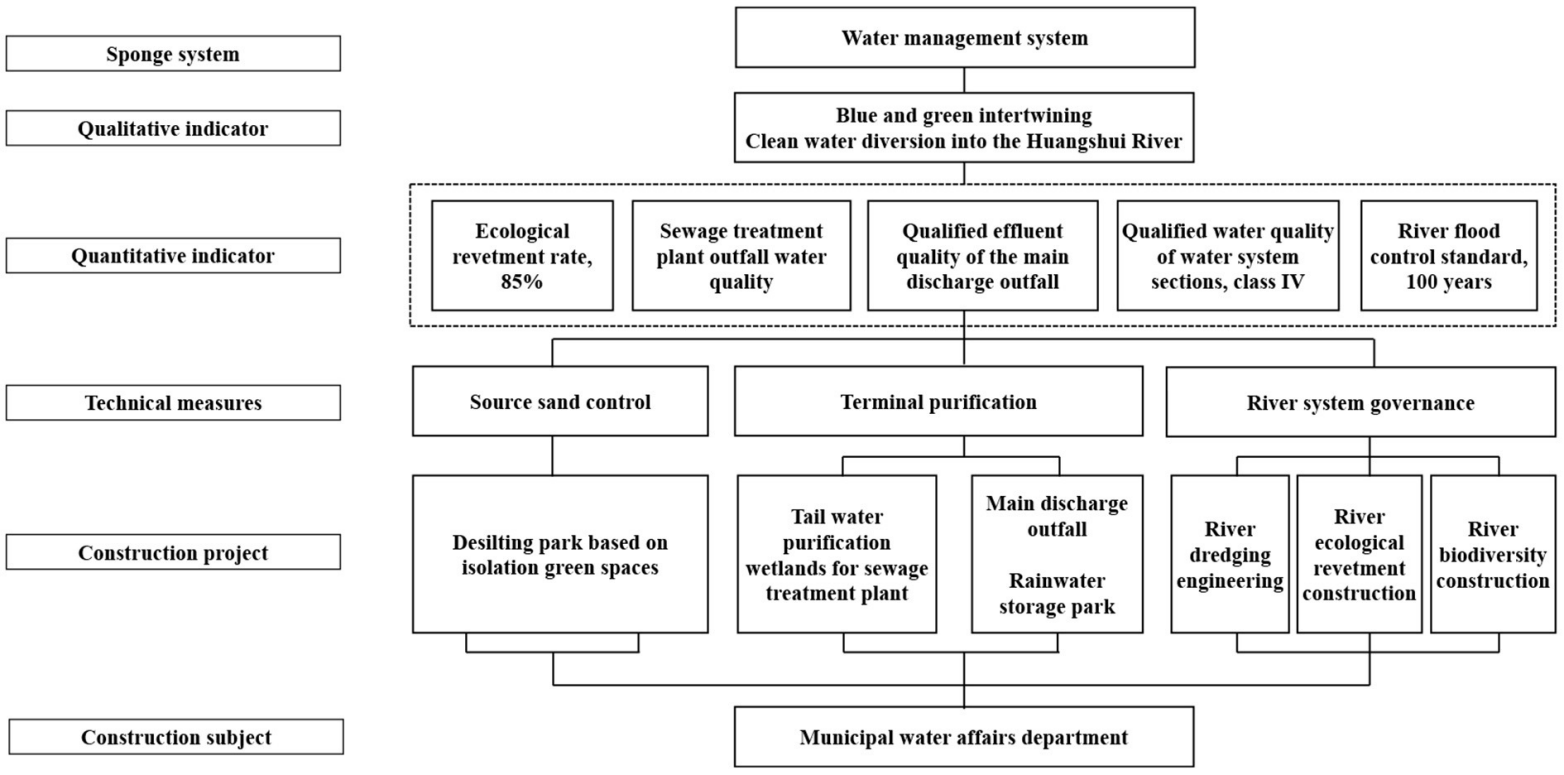
Technical route of water management in the sponge city construction of Xining.

The “water management” system is centered on the main streams in central urban districts. To control exogenous, endogenous, non-point source, and point source pollution and realize the “intertwining of blue and green, and diversion of clean water into the Huangshui River,” source sand control, tail water purification, and river water system control measures are taken. At the upstream, isolation green spaces create a desilting park and guide clean water into the city. By accounting for the location of the sewage treatment plant and the vertical conditions in the urban sections of the river, a wetland park is built for tail water purification with a view. The tailwater of the sewage treatment plant goes through secondary purification to control point source pollution. Moreover, a wetland park is built near the main discharge outfall to control non-point source pollution and provide terminal storm-water storage and purification. Measures such as river dredging engineering, bio-environment creation, and ecological shoreline construction are implemented throughout the river channel to enhance self-purification and realize river ecosystem restoration. The construction projects are integrated into six categories and placed under unified construction and management by the municipal water affairs department. Figure 10 illustrates the technical route of water management.

**Figure 11:**
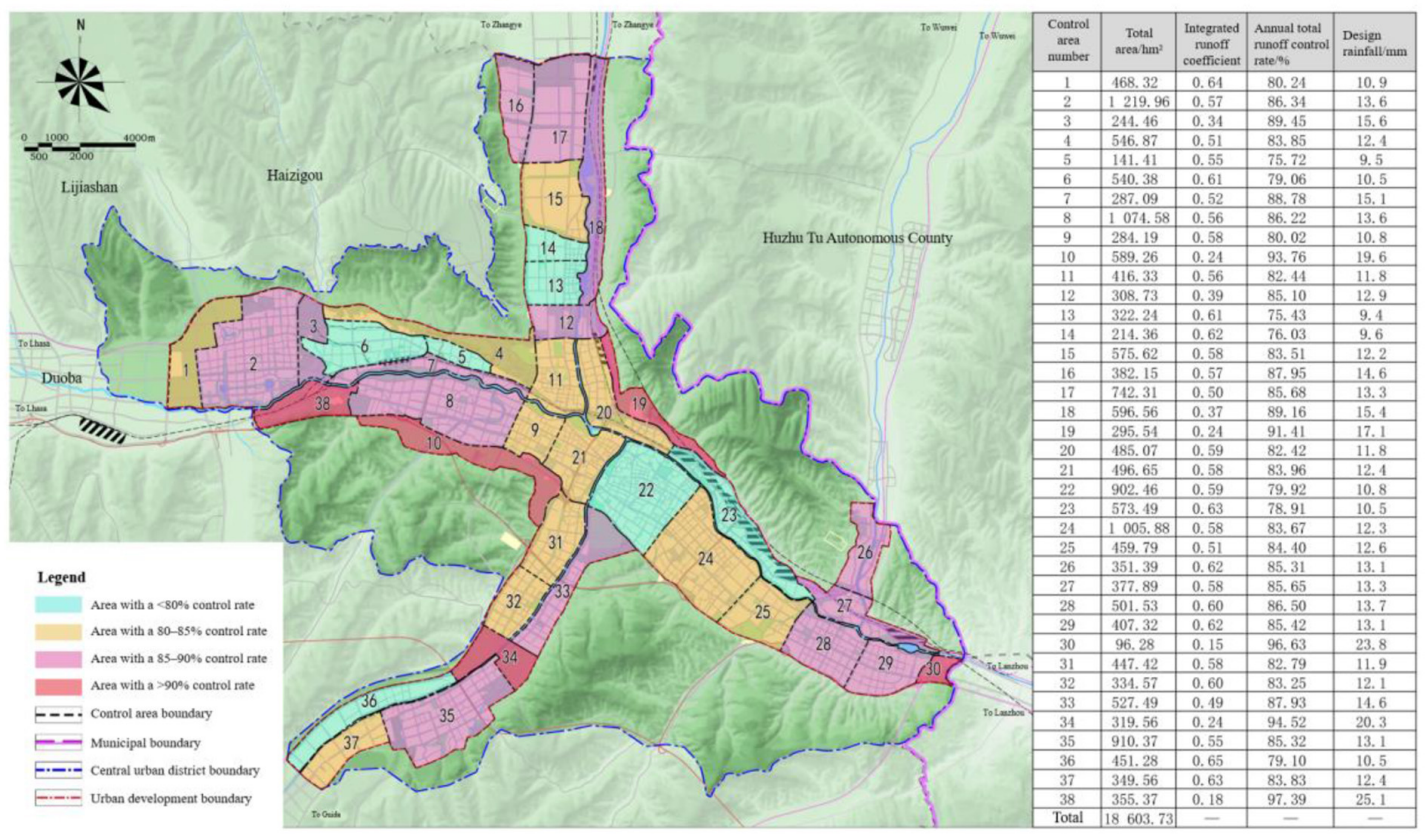
Decomposition of the annual total runoff control rate indicator for the sponge city construction in Xining.

**Figure 12:**
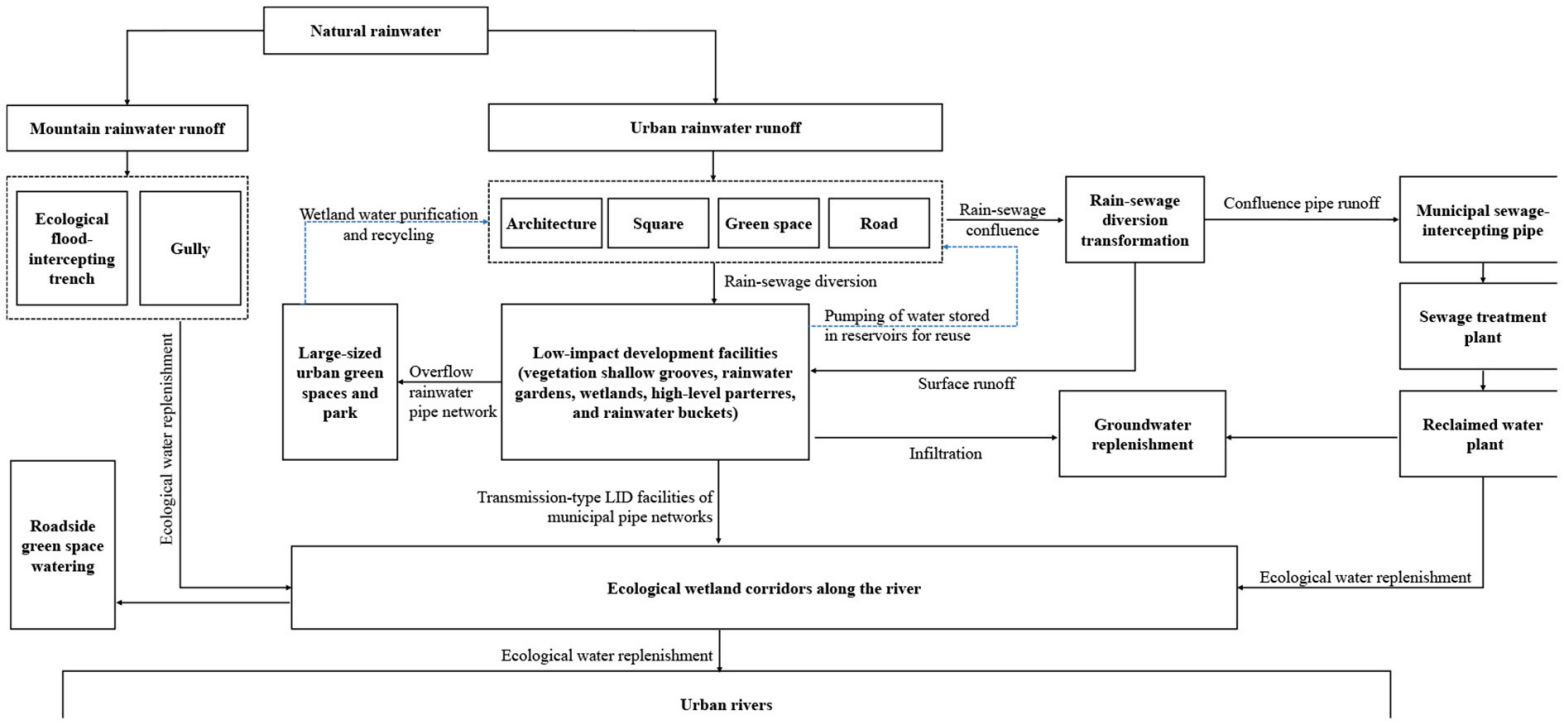
Flow chart of comprehensive rainwater utilization in the sponge city construction of Xining.

Lastly, the “moist city” system is based on the construction land in central urban districts. The goal of “moist city in mild rain, balance of utilization and drainage” is achieved by constructing the source low-impact development system, transforming the midway rain-sewage diversion pipe networks, and building the water resources recycling system. In order to realize the mode transformation from rapid to slow rainwater drainage, low-impact development and source emission reduction system is constructed. The system is characterized by source interception, process guidance, terminal storage, and systematic greening. A small amount of seriously polluted initial rainwater is collected and disposed of to reduce the pollution loads discharged into the municipal river and lake water systems. Figure 11 illustrates the decomposition of the annual total runoff control rate indicator. After this, the locations, pipe lengths, and pipe diameter of combined rainwater pipe networks in the old city are figured out. In principle, the original combined rainwater pipes are used for sewage discharge while the new ones are laid out. In areas faced with land shortages, overflow pollution in rainstorms is prevented by placing sewage interception pipes and setting a sewage interception factor. Finally, to assess the total quantity of available water resources and the shortage of water supply, this study analyzes the balance of water supply and demand in Xining (Table 4). In order to promote the project of “introducing the river of the Datong River into the Huangshui River,” the construction input is increased based on urban water supply engineering planning. The long-term water demand of the city is met by diverting water to the Seventh Water Plant at 800,000 m^3^/d and to the Fifth Water Plant at 25,000 m^3^/d. At the same time, rainwater resource utilization and reclaimed water reuse indicators are allocated in a reasonable manner. In addition, the rainwater utilization system and the reclaimed water recycling system are constructed to encourage the utilization of unconventional water resources. The proportion of rainwater utilized as a substitute for urban tap water has reached 2.6%, while the reclaimed water utilization rate has reached 50%. The reclaimed water is also used as miscellaneous municipal water, ecological landscape irrigation water, and industrial production water, thus promoting the circulation of regional water resources. Figure 12 represents the flow chart of comprehensive rainwater utilization. Finally, it should be noted that the construction projects are integrated into five categories and placed under unified construction and management by the municipal housing and urban-rural development department and water drainage companies.

**Table 4:** Balance of water supply and demand in the central urban districts of Xining.

**Year**	**Water consumption prediction/(10,000 m^3^/d)**	**Water supply scale of existing water plants/(10,000 m^3^/d)**	**Water supply to be added (10,000 m^3^/d)**	**Proportion of added unconventional water utilized as a substitute for urban tap water**	
				**Rainwater**	**Reclaimed water**
2015	44.3	38.6	5.7	0	12.3
2020	55.9	38.6	17.3	1.0	25.9
2030	82.4	38.6	43.8	2.6	29.7

## Conclusions

(1) Based on formulating sponge city special planning in Xining, this paper optimized the “four-water theory.” It made several suggestions for the formulation of sponge city special planning based on the “mountain management–water management–moist city” framework. The aim of these suggestions was to adapt to the semi-arid valley cities in northwestern China. The suggestions made by this study focus on three dimensions: problem identification, indicator system, and planning measures. By introducing the “mountain–water–city” systematic analysis method of supplementing and refining target indicators, this paper constructs the urban sponge system from the perspective of mountains, water systems, and the city. This system also resolves a series of problems with traditional frameworks, such as inadequate problem identification, imperfect indicator system, and overlapping planning measures.

(2) At present, sponge city construction is still in its early development stage in China. The authors propose that sponge city construction should be examined through a developmental perspective while considering the principles of openness and inclusiveness. Furthermore, related technologies should be updated in accordance with practical experience to revitalize the sponge city special planning and better promote sponge cities. The authors hope that this research will provoke meaningful discussions, offer several new suggestions, and raise the overall level of sponge city special planning.

## Data availability statement

The original contributions presented in the study are included in the article/supplementary material, further inquiries can be directed to the corresponding author.

## Author contributions

RW: conceptualization, funding acquisition, supervision, and writing—original draft. YY: funding acquisition, formal analysis, methodology, writing—review, and editing. All authors contributed to the article and approved the submitted version.

## Funding

This study was supported by the National Natural Science Foundation of China Youth Fund (Grant No. 52008346) and the Self-supporting Scientific Research Project of China Urban Construction Research Institute Co., Ltd. (Grant No. Y07Y22007).

## Conflict of interest

Authors YY, JH, and JZ were employed by China Urban Construction Design & Research Institute Co., Ltd. The remaining author declares that the research was conducted in the absence of any commercial or financial relationships that could be construed as a potential conflict of interest.

## Publisher's note

All claims expressed in this article are solely those of the authors and do not necessarily represent those of their affiliated organizations, or those of the publisher, the editors and the reviewers. Any product that may be evaluated in this article, or claim that may be made by its manufacturer, is not guaranteed or endorsed by the publisher.
